# Whole genome sequencing reveals new links between *spa* t172/CC59 methicillin-resistant *Staphylococcus aureus* cases in low-endemicity region of Southwest Finland, 2007‒2016

**DOI:** 10.1038/s41598-022-25556-w

**Published:** 2022-12-09

**Authors:** Jaakko Silvola, Kirsi Gröndahl-Yli-Hannuksela, Tiina Hirvioja, Kaisu Rantakokko-Jalava, Esa Rintala, Kari Auranen, Jenna Junnila, Harri Marttila, Laura Lindholm, Jaana Vuopio

**Affiliations:** 1 Institute of Biomedicine, University of Turku, Turku, Finland; 2 Department of Hospital Hygiene & Infection Control, Turku University Hospital, Turku, Finland; 3 Clinical Microbiology Laboratory, Turku University Hospital, Turku, Finland; 4 Department of Mathematics and Statistics and Department of Clinical Medicine, University of Turku, Turku, Finland; 5 Department of Health Security, Finnish Institute for Health and Welfare, Helsinki, Finland

**Keywords:** Bacteria, Microbiology, Antimicrobial resistance, Molecular medicine

## Abstract

Methicillin-resistant Staphylococcus aureus (MRSA) rates have remained relatively low in Finland. In Southwest Finland, however, annual MRSA incidence increased from 12 to 25/100,000 between 2007 and 2016 with *spa* t172 strain causing one fourth (237/983) of all cases. This provoked us to study the molecular epidemiology of t172-MRSA, aiming to better understand the transmission of this strain type. We combined epidemiological data and whole genome sequencing (WGS) of a set of 64 (27%, 64/237) t172-MRSA isolates covering 10 years. Isolates represented sporadic and index cases of all identified healthcare-associated outbreaks (HAOs) and family clusters (FCs). Among the included 62 isolates, core-genome MLST analysis revealed eight genomic clusters comprising 24 (38.7%) isolates and 38 (61.3%) non-clustered isolates. Cluster 1 comprised ten and the remaining seven clusters two isolates each, respectively. Two epidemiologically distinct HAOs were linked in cluster 1. FCs were involved in all clusters. All strains were associated with epidemic clonal complex CC59. We were able to confirm the spread of several successful t172-MRSA subclones in regional healthcare and the community. WGS complemented routine surveillance by revealing undetected links between t172-MRSA cases. Targeted, WGS-based typing could enhance MRSA surveillance without the need for routine WGS diagnostics.

## Introduction

Methicillin resistant *S. aureus* (MRSA) remains a major public health concern and the burden of disease caused by MRSA is significant^1^. In Europe, the population-weighted mean proportion of MRSA among reported *S. aureus* isolates has been on the decline although the overall incidence of severe *S. aureus* infections has been increasing during the last decade^2,3^. In Finland, the incidence of MRSA has remained relatively low during recent years, although there is interregional variation in the incidence and proportion of *spa* types^4^. A notable increase in the annual MRSA incidence was recently reported in the Hospital District (HD) of Southwest Finland, with *spa* type t172 isolates causing one-fourth of all cases^5^.

*Spa* typing, based on the sequencing of tandem repeat pattern in the staphylococcal *spa* gene, is the routine MRSA strain typing method used in many countries including Finland^6^. *S. aureus* isolates with *spa* t172 are common in Finland while in the other Nordic countries they are among the more infrequently reported strain types^7^. Since the routine adaptation of *spa* typing in 2009, t172-MRSA isolates have consistently remained among the most prevalently reported *spa* types^8,9^. In Finland, strains classified with pulsed-field gel electrophoresis (FIN-4) and multi-locus sequence typing (MLST, ST375), associated later with *spa* t172, were first reported in 1993 and increasingly towards the end of the 1990’s^9–11^. The strain was first detected in several hospital outbreaks although later identified as the leading community-associated MRSA strain^11^. Recently, t172 cases have been reported at the national level in healthcare-associated outbreaks and community-associated infections with varying clinical severity^12,13^. Previously reported phenotypic and genetic characteristic features of the t172 MRSA isolates are susceptibility to non-β-lactam antibiotics, small staphylococcal cassette chromosome *mec* (SCC*mec*) types IV and V, and Panton-Valentine Leucocidin (PVL) negativity^14^.

The aim of this study is to retrospectively elucidate the molecular epidemiology of t172 MRSA reported in the HD of Southwest Finland between 2007 and 2016. By combining epidemiological and cluster analyses based on whole genome sequencing (WGS) we aim to advance the understanding on the demographics, clonality and transmission pattern of t172 MRSA in healthcare and community settings.

## Results

### *Spa* type distribution

**Figure 1:**
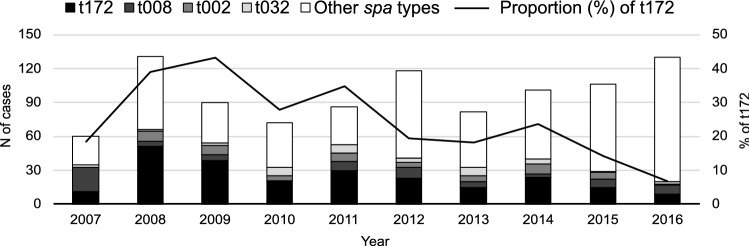
Annual number of MRSA cases caused by the four most common or other *spa* types and the proportion of t172, Hospital District of Southwest Finland, years 2007–2016 (n = 976).

Within our 10-year population-based study 237/976 (24.3%) t172 cases were identified. The most frequent *spa* type was t172, followed by t008 (74/976, 7.6%), t002 (55/976, 5.5%) and t032 (40/976 4.1%) (Fig. 1). Altogether 173 different *spa* types were reported and t172 cases were reported throughout the study period. Their proportion of all MRSA cases peaked in 2009 (43.3%) and decreased towards 2016 (6.9%).

### Demographics and epidemiological classification of t172 cases

**Figure 2:**
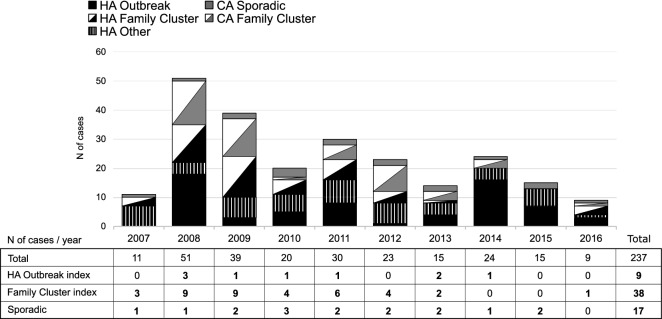
Epidemiological classification and the total number of index/sporadic t172 MRSA cases (n = 273) in the Hospital District of Southwest Finland, years 2007–2016. All t172 index cases and sporadic cases were selected for the WGS based analysis (bold). Healthcare-associated/Community-associated (HA/CA).

Based on the available epidemiological information, t172 cases were distributed between 170 (71.7%) healthcare associated (HA-MRSA) and 67 (28.3%) community associated (CA-MRSA) cases (Fig. 2). Within the HA-MRSA group, 74 cases were associated with healthcare associated outbreaks (HAOs), 51 cases with family clusters (FCs), and 45 were defined as other HA cases. Within the CA-MRSA group, 50 cases were associated with FCs and 17 cases were defined as sporadic.

All t172 index cases of HAOs (n = 9), FCs (n = 38) and all sporadic t172 cases (n = 17) were selected to the WGS analysis. Among all cases, a total of 26 HAOs and 129 FCs were identified while t172 cases were involved in 12 (46.2%) and 39 (30.2%) of these, respectively. In three HAOs and one FC where t172 cases were involved, the index case was other than t172. These four clusters were not included. The selected cases spread throughout the study period (Fig. 2). Additionally, 16 t172 isolates from cases of HAO investigations were used for comparison in the cgMLST cluster analysis.

The median age of the t172 cases was 58 years (range 0.1–100.9 years), which is significantly higher than other *spa* types combined (median 40 years, range 0.1–103.4 years, *p *< 0.001). The t172 cases were not associated unambiguously with either HA- or CA-MRSA groups (Table 1). Within the HA-MRSA group, t172 cases were associated with long-term care and HAOs, whereas association to hospital care abroad was negative. In the CA-MRSA group, t172 cases were negatively associated with sporadicity. Additionally, t172 cases were associated with FCs, but association with immigrant status, livestock contact and travel or working abroad was negative. No differences between t172 and other *spa* type cases were observed in terms of sex, history of intravenous drug use, HCW status or sample type.

**Table 1:** Demographic and epidemiological characteristics of t172 and other *spa* type MRSA cases, Hospital District of Southwest Finland, years 2007–2016.

Variable	Total n (%)	*spa* t172 cases n (%)	Other *spa* type cases n (%)	*p*^*d*^
Case count	976	237 (24.3)	739 (75.7)	–
Median age in years, IQR	43.8, 19.4–68.2	58.4, 34.2–82.6	39.6, 17.1–62.2	**< 0.001**
Male sex	442 (45.3)	116 (48.9)	326 (44.1)	0.203
Screening sample	702 (74.2)	177 (74.7)	525 (71.0)	0.319
CA-MRSA	307 (31.5)	67 (28.3)	240 (32.5)	0.260
Sporadic cases	143 (14.7)	17 (7.2)	126 (17.1)	**< 0.001**
HA-MRSA	669 (68.5)	170 (71.7)	499 (67.5)	0.260
Long-term care residence	175 (17.9)	55 (23.2)	120 (16.2)	**0.019**
HCW	69 (7.1)	15 (6.3)	54 (7.3)	0.494
Hospital care abroad^a^	155 (15.9)	12 (5.1)	143 (19.4)	**< 0.001**
Link to HAO	223 (22.8)	74 (31.2)	149 (20.2)	**0.001**
Link to FC	321 (32.9)	101 (42.6)	220 (29.8)	**< 0.001**
**Other identified risk groups**				
Immigrant status^b^	209 (21.4)	8 (3.4)	201 (27.2)	**< 0.001**
Travel or work abroad^c^	237 (24.3)	5 (2.1)	83 (11.2)	**< 0.001**
Live-stock contact	22 (2.3)	0 (0)	22 (3.0)	**0.004**
Intravenous drug use	6 (0.6)	1 (0.4)	5 (0.7)	–

^a^Within previous two years ^b^Asylum seeker, refugee, resident of other country or other immigrant ^c^Within the previous year ^d^Fisher exact test or Mann–Whitney U test where appropriate ^d^Statistical significance *p* < 0.05.

*IQR* Interquartile range, *HCW* Healthcare worker, *CA/HA-MRSA* Community-associated/Healthcare-associated Methicillin resistant *S. aureus*, *FC* Family cluster, *HAO* Healthcare-associated outbreak.

Significant values are in [bold].

### WGS based analysis

In total, 64 t172 MRSA isolates representing the index cases of the HAOs (n = 9) or FCs (n = 38) and all sporadic cases (n = 17) were selected for WGS. After excluding two samples (CA-MRSA, sporadic case, year 2016: isolate not available, and HA-MRSA, FC index case, year 2013: low quality in WGS), altogether 62 samples were included in the WGS based analyses. An average of 125-fold coverage (range 75–183) and N50 of 79,280 were observed. Sixty isolates were ST375 and two were single locus variants (SLVs) of ST375 (ST59 and ST5428, Table S1). Predicted SCC*mec* elements included indication of SCC*mec* type IV(2B) in 60 isolates and type V(5C2&5) in two (SWF_2 and SWF_13) isolates. No discrepancy between WGS based and conventional *spa* typing was detected. Three technical control duplicate sequences from THL confirmed identical typing results in core-genome multi-locus sequence typing (cgMLST) between the laboratories.

**Figure 3:**
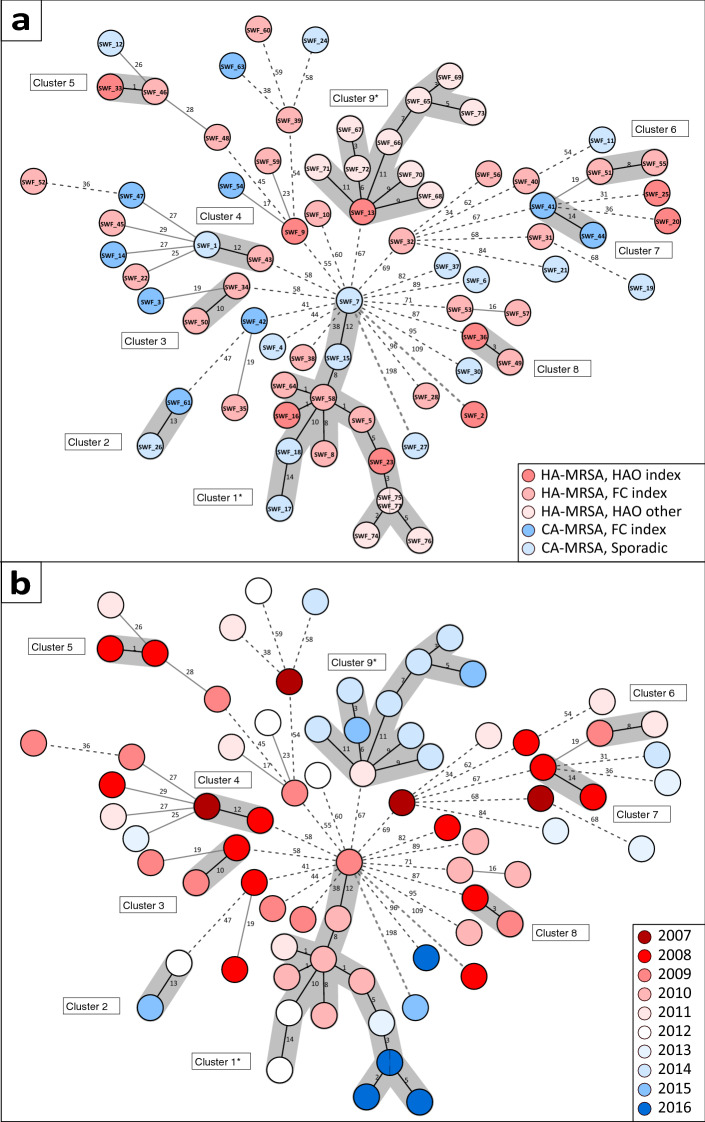
**(a)**Minimum spanning tree based on single nucleotide variants (SNVs) between the core-genome alleles of 75 *spa* t172 MRSA isolates, Hospital District of Southwest Finland, years 2007–2016. Each node represents one or two isolates as stated in the node. 62 isolates represent index cases from healthcare-associated outbreaks (HAO, n = 9) and family clusters (FC, n = 37) and sporadic cases (n = 16). The rest, 13 isolates, are from other additional HAO cases acquired via clinical HAO investigations. Clusters are highlighted with grey background, distance threshold 15 SNVs. Epidemiological case data shown by the colour scheme as stated in the figure legends: blue indicates community (CA-MRSA) origin, with additional parameters: dark blue family cluster (FC) index case and light blue sporadic case. Red indicates healthcare (HA-MRSA) origin: dark red HAO index case and, light red FC index case and pale red other HAO case. (**b)** Isolate detection year shown by the colour scheme. *Clusters including isolates from clinical HAO investigations (legend: HAO other) marked with asterisk.

In cgMLST based analysis, the pairwise median single-nucleotide variant (SNV) distance was 75 (range 1–242). Eight genetically distinct clusters were identified, comprising 24 (38.7%) isolates, while 38 (61.3%) isolates did not cluster (Fig. 3a). One cluster (cluster 1, Fig. 3) comprised 10 isolates and the other seven clusters comprised two isolates each. Altogether 12 (19.4%) of the clustered isolates and 17 (27.4%) of the non-clustered isolates were from clinical MRSA infections and other 33 (53.2%) isolates from screening samples with an identified risk factor (Table S1). Additional HAO investigation isolates were incorporated to cluster 1 (3 isolates) and formed an individual cluster (cluster 9, 10 isolates, Fig. 3).

The ten isolates belonging to the largest cluster (cluster 1) represented variable epidemiological backgrounds. The earliest case linked to cluster 1 was a sporadic CA-MRSA case isolated in 2009. Three other sporadic CA-MRSA cases in the cluster were isolated in 2010 and 2012. Six other cases in cluster 1 were HA-MRSA. Of these, two were HAO index cases. Interestingly, these two separately perceived HAOs were revealed to share the same genomic background. These HAOs took place in 2009–2012 (index SWF_16) and 2013–2016 (index SWF_23), respectively. Other four HA-MRSA cases in cluster 1 were from FC index cases isolated in 2010 and 2011. Based on the epidemiological data, 3/4 of these FC index cases were previously linked to the earlier HAO (index SWF_16) in cluster 1 and thus WGS supported these linkages (Table S1).

Clusters 2–8 comprised two isolates each. All these clusters involved a FC index case and revealed previously undetected links between FCs, HAOs and sporadic cases (Fig. 3a). In clusters 2 and 4, a FC index case and a sporadic case were linked. In clusters 3, 6 and 7, two separate FC index cases were linked. In clusters 5 and 8, a FC index case and a HAO index case were linked. In cluster 5 there was also a link between the cases based on the epidemiological data and thus WGS supported the link. There were no other previously identified epidemiological links in clusters 2–8. In all clusters with HAO index case involved (clusters 1, 5 and 8), isolation dates suggest transmission direction from healthcare to households (Fig. 3b).

The non-clustered isolates (n = 38, 61.3%) were distributed in the population with 16–198 SNVs distance to their genetically closest isolate (Fig. 3). Three additional and two extended clusters would have been formed if we had used a slightly wider clustering cutoff (16 to 19 SNVs, Fig. 3). Otherwise, the dispersion of small clusters in the population was clear (25 or more SNVs to the closest isolate). Only two isolates (HAO index SWF_2 and sporadic SWF_27) differed with over 100 SNVs to their closest isolate. The non-clustered isolates represented 10 sporadic, 23 FC and 5 HAO index cases. These isolates were reported throughout the whole study period. Based on the epidemiological data, one FC index case (SWF_52) was linked to a HAO (index SWF_9), but interestlingy, based on the WGS analysis, they did not share genomic background (pairwise distance 72 SNVs) refuting probable transmission. No other apparent epidemiological links were refuted in the WGS analysis (Table S1).

### Additional isolates

In this study, additional WGS sequence data from 16 t172 MRSA isolates from three separate t172 HAO investigations (index cases SWF_2, SWF_13 and SWF_23) were included in the cgMLST analysis as technical controls (n = 3) and for comparison (n = 13) of knowingly clustered isolates. From these 13 additional isolates from two different HAO investigations, three isolates were incorporated to one of the previously mentioned clusters (cluster 1) and 10 isolates formed one additional cluster (cluster 9, Fig. 3). These additional isolates demonstrate how conventional outbreak-based investigations relate to our selection of the local t172 MRSA strains. As seen in cluster 1, not all cases associated with the outbreak were previously detected as they had been isolated up to 7 years before the HAO investigation in 2016 (Fig. 3b). However, cluster 9 included only cases epidemiologically recognized in the HAO investigation in 2015.

## Discussion

This study characterized the demographics and molecular epidemiology of t172 MRSA cases in the HD of Southwest Finland during years 2007–2016. Demographic results show, that the t172 cases represented diverse epidemiological groups while a strong indication of primarily domestic transmission was confirmed. Combining epidemiology and a targeted WGS analysis, we were able to assess the clonality of the strains and detect new transmission clusters. Seven new transmission clusters were detected, one confirmed and one refuted using WGS and a diverse t172-MRSA background population was revealed.

In this study, the demographic features of t172 MRSA reflect the domestic nature of transmission, which is also supported by the low reporting frequency of t172 elsewhere in the world (Table 1)^7,9^. The increase of *spa* type diversity and the decreasing proportion of t172 in the HD of Southwest Finland were detected simultaneously with the increase in the incidence of cases with immigrant status and hospital care abroad as well as a switch towards younger age groups^5^. Especially the increasing prevalence of *spa* t304 strains in many European countries has been discussed in the context of refuge and immigration from the Middle-East (Iraq, Syria) during the last decade^15,16^. However, t172 has been continuously reported nationally and has been only partially replaced in prevalence by other *spa* types by the end of 2021 (4th most prevalent *spa* type, 5% of all Finnish MRSA cases)^4^. Our results also show higher median age and stronger association with both local household and healthcare outbreaks among the t172 cases (Table 1). Therefore, the observed differences in the epidemiology of MRSA strains would argue against the assumption that foreign influx and direct replacement of strain types via competition are solely behind the gradual local decrease in t172 cases. Other explanations possibly contributing to the decline of t172 are changing environmental factors such as infection control practices and changes in the screening schema^17^.

Although t172 strains are infrequently reported outside Finland, related strains are common elsewhere. The single locus variant strain types of ST59, such as ST375 associated with t172, belong to clonal complex CC59. It is a globally successful CA-MRSA lineage with major East-Asian and North-American subclades, which are both reported in Europe^18,19^. In China, CC59 has become one of the dominant lineages in many hospitals^20^. Additionally, CC59 strains have been reported in western Australia, Japan and are among the most prevalent strains in Sweden, Norway and Iceland^7,21,22^. CC59 strains appear to be genetically distinct and not limited to specific regions or host-environments, and generally possess the qualities of high-risk CA-MRSA clones^23,24^. The clonal nature and success of t172/CC59 strain is reflected in our results in its tendency of forming distinct and persistent genetic clusters between community and hospital environments. It is also reflected in the shared CC59 genotype and associated SCC*mec* elements^18,20^. In the future, a phylogenomic analysis using genomic sequences from a representative take of international CC59 MRSA isolates would help to understand the route of dissemination and clonal origin of t172/CC59 more deeply.

Previously, t172 strains has been isolated from asymptomatic carriers as well as from severe MRSA/MSSA infections^13^. The majority of t172 isolates in our study were from screening samples which reflects the role of asymptomatic carriers in CA-MRSA transmission as well as the MRSA screening policy in the HD^5^. Invasive MRSA infections in the HD are rare and during 2007–2016 only 10 MRSA isolates were reported from bloodstream infections^25^. Data on sample types in this study were gathered by combining all screening and clinical infection samples and the detailed information about the culture/specimen type was defective. Thus, only five of the above mentioned blood culture isolates were identified and interestingly, one strain from these was t172.

A major proportion of all t172 cases (42.6%) in this study were linked with FCs. In the WGS analysis, all the identified clusters included a FC index case. The role of households in CA-MRSA transmission is acknowledged and household transmission has been recognized as a major source of new MRSA acquisitions in low-prevalence settings^26–28^. Our findings reflect the potential of t172 MRSA strains to persist and spread between different environments, a commonly reported observation in epidemiological studies involving CA-MRSA^29^. Although dichotomous HA-/CA-MRSA classification has been questioned, HA-MRSA strains seem to spread more limitedly outside healthcare settings while CA-MRSA clones are commonly reported in healthcare^29–32^. In addition to detecting transmission clusters, the WGS analysis both confirmed (cluster 9) and refuted (SWF_9, SWF_52) epidemiological links between t172 cases. Importantly, in cluster 1 two epidemiologically distinct HAOs were linked via FC index cases and seven out of eight clusters we identified would not have been noticed at all as part of conventional surveillance due to the temporal distance and lack of epidemiological link between the cases. The resolution of *spa* typing combined with extensive epidemiological tracing was thus insufficient to distinguish certain outbreaks. Therefore, targeted sequencing strategy of isolates from a limited number of epidemiological groups of known risk (*e.g.* secondary cases of FCs linked by WGS) can be a potential strategy when trying to limit the spread of certain MRSA strains, such as t172, without the need for routine WGS diagnostics^33^.

WGS has been used widely to confirm, detect or refute hospital outbreaks and previously undetected transmission of MRSA^34^. Although the cost and lack of global nomenclature hinders its adoption as routine diagnostics, software development has extended the use of WGS^35^. Several allelic and SNV cutoffs for clustering closely related MRSA strains have been discussed in the literature depending on the method. Strains with strong epidemiological links tend to differ from each other by under 20 core genome SNVs^28,33,36–38^. Although our approach was sample-centric and based on a previously defined core-genome, we chose to use a conservative 15 core-genome SNV cutoff proposed recently by Coll F. *et al*^39^. They analyzed over 1000 MRSA cases and concluded 15 core-genome SNVs as the best genetic cutoff to rule out transmission events up to 6 months. This means that some putative transmission events (16 to 20 SNVs) in our data may have been overlooked, as seen between some isolates and two WGS clusters (clusters 6 and 7, Fig. 3). Nevertheless, the use of a stringent SNV cutoff increases the credibility of identified clusters. The isolation dates of clustered isolates ranged from less than a year up to four years, which suggests a local long-term reservoir of clonal t172 strains (Figre 3b). Overall, it can be concluded that the genetic distance of the isolates in this small selection reflects the circulating population of t172 MRSA in the HD, where some subclones have been successful in causing outbreaks despite active preventive measures^20,40–42^.

There are some limitations in our study. The number of isolates included in the WGS analysis limits the power of the study. Carefully defined criteria, however, were used when selecting the isolates to ensure epidemiological diversity during the 10-year study period, and a conservative SNV cutoff was used to avoid overestimation of identity. The availability of extensive epidemiological register data is a strength of this study.

Based on our results, we conclude that t172 MRSA strains can spread between different environments in the community as well as in hospitals and cause persistent hospital outbreaks in a setting with generally low endemicity of MRSA. The t172 strain spread among older patients and was not introduced by patients with foreign healthcare contacts or origin. Endemic transmission of high-risk CA-MRSA strains can be challenging to prevent, which is reflected in the major role of FCs and asymptomatic carriers. Despite the cost-effective detection of diversity among MRSA strains with *spa* typing, the resolution might be insufficient when looking at transmission links on the individual level. The potential of WGS in outbreak investigations is well established, however, an integrated approach could provide a useful and cost-effective way of tracking high-risk MRSA transmission^33^. Our results encourage development of targeted WGS based MRSA surveillance to accompany screening and risk factor identification to improve detection and prevention of persistent MRSA clone circulation.

## Methods

### Study material and definitions

The *spa* t172 MRSA cases and the respective bacterial isolates were identified through our previous retrospective, population-based study covering all 983 new MRSA cases in the HD of Southwest Finland (catchment population of 478 500, representing 8.7% of the Finnish population in 2016) between 2007 and 2016. Seven cases lacking data on the *spa* type were excluded (n = 976)^5^. No informed consent was asked from the subjects due to the retrospective, register-based nature of the study. All experiments and analyses were performed in accordance with relevant guidelines and regulations.

For each case, the following demographic and background data on possible risk factors were available: age, sex, specimen type (screen/clinical MRSA infection), immigrant status, live-stock contact, intravenous drug use, long-term care residence, healthcare worker (HCW) status, travel or work abroad within the previous year, and hospital care abroad within the previous 2 years. All cases were defined as either healthcare (HA-MRSA), or community associated (CA-MRSA). Cases in HCWs, infants under 28 days of age and patients who had been hospitalized (including long-term facilities) within the last two years in Finland or the previous year abroad were defined as HA-MRSA. Other cases were defined CA-MRSA. HAOs, including hospital and long-term facility outbreaks, were defined as at least two cases in HCWs or patients sharing a room/washing facility in the same unit. FCs were defined as two or more cases living in the same household. Index cases were defined as the first identified case leading to an investigation of respective HAO or FC. Additionally, a case was defined sporadic if it lacked an epidemiological link to healthcare or FCs. HA-MRSA cases without a link to HAO or FC were defined as other HA cases. This detailed epidemiological information was acquired via systematic epidemiological tracking of cases, performed by the local infection control unit.

Currently, all Finnish MRSA isolates are *spa* typed by The Finnish Institute for Health and Welfare (THL) while HAOs are investigated with WGS upon request^7^. THL reports the typing results to the referring clinical laboratory and the infection control unit of the HD. In this study, isolates from all sporadic t172 MRSA cases and index cases of FCs and HAOs from the HD were chosen for the WGS-based analyses. All existing epidemiological links between the cases were identified before WGS clustering (Table S1). Additional WGS data gathered through three HAO investigations in 2008, 2011–2015 and 2013–2016 (performed by THL) of 16 isolates were included as technical controls (3/16) and comparison (13/16). All cases were pseudonymized and an arbitrary nomenclature for study isolates was generated (SWF number).

### Whole genome sequencing

All isolates were sequenced on MiSeq platform (Illumina, USA) using V2 chemistry with 250 bp paired-end reads. Chromosomal DNA was isolated with MagAttract HMW DNA kit (Qiagen, Germany) according to the manufacturer’s instructions. DNA concentrations were normalized with Qubit fluorometer (Invitrogen, United Kingdom). Libraries were prepared with Nextera XT DNA sample preparation kit (Illumina, USA) and multiplexed to 22 isolates per run.

SeqSphere + v.7.2.3 (Ridom Gmbh) pipeline was used for sequence analysis including data quality and adapter control (FastQC) and de novo assembly with SKESA v.2.3^43^. A public cgMLST task template with 1861 targets for *S. aureus* was used with allele calling parameters as described previously^44^. Isolates with < 95% good targets in cgMLST were excluded. Additionally, a sample-centric SNV search, with insertion/deletion filtering, was used to generate a minimum-spanning tree (MST) based on the nucleotides of found SNV positions between samples. Pairwise ignoring of missing value option was chosen and cluster distance cutoff was set at 15 SNVs^39^. STs and *spa* types were called from sequencing data as implemented in SeqSphere + . SCC*mec* elements were predicted using a web-based tool SCCmecFinder v.1.2 assuming the cassette with best homology when missing genes/complexes were reported^45^. All genome sequence data have been deposited into the National Center for Biotechnology Information Sequence Read Archive under BioProject accession no. PRJNA826681.

### Statistical analysis

Statistical analyses were conducted with SPSS v.25 software (IBM SPSS, Armonk, NY, USA). Statistical significance was determined as *p* < 0.05. Descriptive statistics are reported as the number and proportion of positive observations or median and interquartile range (IQR) as appropriate. Demographic binary variables between t172 and other MRSA cases were compared pair-wise using Fisher’s exact test reporting two-sided *p-*values. Mann–Whitney U test was used for non-normally distributed outcomes.

### Ethics approval

The study was approved by the Hospital District of Southwest Finland (T162/2016; J28/21), and the Finnish Institute for Health and Welfare (THL336/6.02.00/2016; THL/319/5.05.00/2020). According to the Finnish Medical Research Act (488/1999), Act of the Medical Use of Human Organs, Tissues and Cells (101/2001) and Biobank Act (688/2012) as amended, and as confirmed by the Hospital District of Southwest Finland Research Ethics Committee, no ethical committee approvals or informed consent were needed for this retrospective, register-based study.

## Supplementary Information

Supplementary Information.

## Data Availability

The datasets generated during the current study are not publicly available as they contain health related data but limited datasets (without any identifiable, person-related data) are available from the corresponding author on reasonable request. All genome sequence data have been deposited into the National Center for Biotechnology Information Sequence Read Archive under BioProject accession no. PRJNA826681.
